# IRBIT Interacts with the Catalytic Core of Phosphatidylinositol Phosphate Kinase Type Iα and IIα through Conserved Catalytic Aspartate Residues

**DOI:** 10.1371/journal.pone.0141569

**Published:** 2015-10-28

**Authors:** Hideaki Ando, Matsumi Hirose, Laura Gainche, Katsuhiro Kawaai, Benjamin Bonneau, Takeshi Ijuin, Toshiki Itoh, Tadaomi Takenawa, Katsuhiko Mikoshiba

**Affiliations:** 1 Laboratory for Developmental Neurobiology, RIKEN Brain Science Institute, Wako, Saitama, Japan; 2 Division of Biochemistry, Kobe University Graduate School of Medicine, Kobe, Hyogo, Japan; 3 Biosignal Research Center, Organization of Advanced Science and Technology, Kobe University, Kobe, Hyogo, Japan; Institut de Génétique et Développement de Rennes, FRANCE

## Abstract

Phosphatidylinositol phosphate kinases (PIPKs) are lipid kinases that generate phosphatidylinositol 4,5-bisphosphate (PI(4,5)P_2_), a critical lipid signaling molecule that regulates diverse cellular functions, including the activities of membrane channels and transporters. IRBIT (IP_3_ R-binding protein released with inositol 1,4,5-trisphosphate) is a multifunctional protein that regulates diverse target proteins. Here, we report that IRBIT forms signaling complexes with members of the PIPK family. IRBIT bound to all PIPK isoforms in heterologous expression systems and specifically interacted with PIPK type Iα (PIPKIα) and type IIα (PIPKIIα) in mouse cerebellum. Site-directed mutagenesis revealed that two conserved catalytic aspartate residues of PIPKIα and PIPKIIα are involved in the interaction with IRBIT. Furthermore, phosphatidylinositol 4-phosphate, Mg^2+^, and/or ATP interfered with the interaction, suggesting that IRBIT interacts with catalytic cores of PIPKs. Mutations of phosphorylation sites in the serine-rich region of IRBIT affected the selectivity of its interaction with PIPKIα and PIPKIIα. The structural flexibility of the serine-rich region, located in the intrinsically disordered protein region, is assumed to underlie the mechanism of this interaction. Furthermore, in vitro binding experiments and immunocytochemistry suggest that IRBIT and PIPKIα interact with the Na^+^/HCO_3_^−^ cotransporter NBCe1-B. These results suggest that IRBIT forms signaling complexes with PIPKIα and NBCe1-B, whose activity is regulated by PI(4,5)P_2_.

## Introduction

The hydrolysis of phosphatidylinositol 4,5-bisphosphate (PI(4,5)P_2_) in response to the activation of cell surface receptors generates inositol 1,4,5-trisphosphate (IP_3_), which then activates IP_3_ receptors (IP_3_Rs) that are intracellular Ca^2+^ channels located mainly on the endoplasmic reticulum [1]. We previously identified an IP_3_R-binding protein termed IRBIT (IP_3_ R-binding protein released with inositol 1,4,5-trisphosphate) from rat cerebellum [2]. IRBIT binds to the IP_3_-binding domain of IP_3_R through amino acids that interact with IP_3_, and prevents the activation of IP_3_R by competitively blocking access of IP_3_ to IP_3_R [3–5]. IRBIT comprises an N-terminal domain containing a serine-rich region and a C-terminal domain homologous to methylation pathway enzyme S-adenosylhomocysteine hydrolase (AHCY) [2, 6]. The serine-rich region of IRBIT contains multiple phosphorylation sites [3, 6–8]. Priming phosphorylation at Ser68 induces sequential phosphorylation at Ser71, Ser74, and Ser77 by casein kinase I, which requires a priming phosphate located three amino acids upstream of the target serine/threonine [3, 7]. The phosphorylations of these serine residues are critical for the interaction with IP_3_R [3, 7]. Thr82, Ser84, and Ser85 are phosphorylated in mouse brain [8], however, their functional significance remain unknown.

IRBIT is a multifunctional protein that regulates several target proteins [6], including the Na^+^/HCO_3_^−^ cotransporters NBCe1-B [9–14] and NBCn1-A [13], the Na^+^/H^+^ exchanger NHE3 [15–17], cystic fibrosis transmembrane conductance regulator [10, 18, 19], the Cl^−^/HCO_3_^−^ exchanger Slc26a6 [19], Fip1 [20], ribonucleotide reductase [21], and calcium/calmodulin-dependent protein kinase IIα [22]. In most cases, interactions between IRBIT and these proteins depend on positively charged amino acids in the target proteins [3, 6, 13]; however, the molecular mechanisms by which IRBIT interacts with such diverse proteins remain largely unknown. IRBIT knockout mice show defects in secretion from pancreatic ducts [19] and behavioral abnormalities [22], suggesting multiple IRBIT functions in vivo.

Phosphatidylinositol phosphate kinases (PIPKs) are lipid kinases responsible for the production of PI(4,5)P_2_, an important lipid signaling molecule that regulates numerous cellular processes, including cytoskeletal assembly, endocytosis/exocytosis, vesicular trafficking, cell migration, and ion channel and transporter functions [23–26]. The PIPK family is divided into two main subfamilies with distinct substrate specificities. Type I PIPKs, including PIPKIα [27, 28], PIPKIβ [27, 28], and PIPKIγ [29], phosphorylate phosphatidylinositol 4-phosphate (PI4P) at the 5 position of the inositol ring, whereas type II PIPKs, including PIPKIIα [30], PIPKIIβ [31], and PIPKIIγ [32], phosphorylate phosphatidylinositol 5-phosphate (PI5P) at the 4 position of the inositol ring [33]. PIPKs share a common structure, with a conserved kinase domain and divergent N- and C-termini [25, 26]. Intracellular localization of PIPKs is regulated in part by protein-protein interactions that include associations with PI(4,5)P_2_ effectors. Thus, the synthesis of PI(4,5)P_2_ is coupled to its utilization [24, 34].

To gain further insight into the function of IRBIT, we previously performed proteomic analysis of IRBIT-interacting proteins [20]. One of these proteins belongs to the PIPK family. As described above, PI(4,5)P_2_ produced by PIPKs functions as a precursor of IP_3_, an agonist of IP_3_R. In addition, PI(4,5)P_2_ regulates the activity of NBCe1-B [13, 35]. These relationships between PI(4,5)P_2_ and IRBIT-binding proteins suggest that IRBIT is involved in forming signaling complexes containing PIPKs and PI(4,5)P_2_ effectors. In this study, we analyzed the interactions of IRBIT with the PIPK family. We determined the isoform specificity of the interaction between IRBIT and PIPK family members. We elucidated the intriguing molecular mechanisms by which the phosphorylation sites of IRBIT bind to the catalytic core of PIPKs. Furthermore, we described potential signaling complexes containing IRBIT, PIPKs, and NBCe1-B.

## Materials and Methods

### Plasmids

Hemagglutinin (HA)-tagged IRBIT and its site-directed mutants were described previously [3]. Deletion mutants of IRBIT were generated by subcloning truncated cDNA fragments of IRBIT into pHM6 (Boehringer Mannheim). pcDNA3-IRBIT was generated by subcloning the cDNA encoding mouse IRBIT into pcDNA3 (Invitrogen). Mouse PIPKIα, PIPKIβ, and PIPKIγ, and rat PIPKIIα, PIPKIIβ, and PIPKIIγ were cloned into pEF-Bos-myc mammalian expression vectors as described previously [36]. The nomenclature of PIPKIα and PIPKIβ are different in mouse and human [27, 28] and we use the human nomenclature in this study according to the revised nomenclature in the current GenBank database. Site-directed mutants of PIPKIα and PIPKIIα were generated using QuikChange II Site-Directed Mutagenesis Kit (Stratagene). Deletion mutants of PIPKIα and PIPKIIα were generated by subcloning truncated cDNA fragments of PIPKIα and PIPKIIα into pEF-Bos-myc. Bacterial expression vector encoding PIPKIα and PIPKIIα fused to glutathione S-transferase (GST) was generated by subcloning cDNA encoding PIPKIα and PIPKIIα into pGEX-4T-1 (GE healthcare). Green fluorescent protein (GFP)-tagged NBCe1-B was described previously [9].

### Antibodies

Rabbit anti-IRBIT antibody was described previously [2]. Mouse anti-myc (9E10), goat anti-PIPKIα (C-17), anti-PIPKIβ (D-19), anti-PIPKIγ (M-19), anti-PIPKIIα (N-19), anti-PIPKIIβ (A-15), and control goat IgG were obtained from Santa Cruz Biotechnology. Rat anti-HA (3F10) and mouse anti-myc antibody conjugated with peroxidase were obtained from Roche Applied Science. Donkey anti-rabbit IgG conjugated with horseradish peroxidase (HRP) and goat anti-rat IgG conjugated with HRP were obtained from GE healthcare. Rabbit anti-goat IgG conjugated with HRP was obtained from Medical & Biological Laboratories. Rabbit anti-HA, Alexa Fluor 594-conjugated goat anti-rabbit IgG, and Alexa Fluor 488 or Alexa Fluor 647-conjugated goat anti-mouse IgG were obtained from Invitrogen.

### Cell culture and transfection

Mouse embryonic fibroblast (MEF) cells derived from IRBIT knockout mice were described previously [22]. MEF cells, COS-7 cells, and HeLa cells were maintained in Dulbecco’s modified Eagle’s medium (Nacalai Tesque) supplemented with 10% fetal bovine serum (Equitech-Bio), 50 units/ml penicillin, and 50 μg/ml streptomycin (Nacalai Tesque) in a 5% CO_2_ incubator at 37°C. Transfection was performed at 20%–30% confluency using TransIT transfection reagents (Mirus) according to manufactures’ instruction.

### Immunoprecipitation

Fifteen to eighteen hours after transfection, COS-7 cells were lysed in lysis buffer (10 mM Hepes [pH 7.4], 100 mM NaCl, 2 mM EDTA, and 1% Nonidet P-40) for 30 min at 4°C, followed by centrifugation (20,000 x g, 30 min). The supernatants were incubated with protein G-Sepharose beads (GE healthcare) for 1 hr at 4°C to clarify nonspecific binding to the beads. The clarified supernatants were incubated with 0.5 μg anti-myc or 0.2 μg anti-HA antibody for 1 hr at 4°C, and immune complex was isolated by the addition of 5 μl protein G-Sepharose beads for 1hr at 4°C. The complex was spun down and washed three times with lysis buffer. Precipitated proteins were eluted by boiling in SDS-PAGE sample buffer and analyzed by immunoblotting with appropriate antibodies.

For immunoprecipitation from cerebellum, adult mouse cerebella were homogenized in 9 volumes of 10 mM Hepes buffer (pH 7.4) containing 320 mM sucrose, 2 mM EDTA, 1 mM 2-mercaptoethanol, and protease inhibitor cocktail (Roche Applied Science) with a glass-Teflon homogenizer (950 rpm, 10 strokes). The homogenate was centrifuged at 1,000 × g for 10 min, and the supernatant was centrifuged at 100,000 × g for 30 min to obtain cytosolic fraction (the supernatant) and membrane fraction (the pellet). The membrane fraction was solubilized in 50 mM Hepes, 2 mM EDTA, 1% Nonidet P-40, and protease inhibitors for 30 min at 4°C, followed by centrifugation (20,000 x g, 30 min). The supernatant was added with 100 mM NaCl and diluted two times with immunoprecipitation buffer (10 mM Hepes [pH 7.4], 100 mM NaCl, 2 mM EDTA, and 0.5% Nonidet P-40). The cytosolic and the solubilized membrane fractions were pre-cleared with protein G-Sepharose beads for 1 hr at 4°C, and the clarified supernatants were incubated with 2 μg anti-PIPK or control antibody pre-bound to 5 μl protein G-Sepharose beads for 2 hr at 4°C. The complex was spun down and washed three times with immunoprecipitation buffer. All animal studies were carried out in accordance with the guidelines and approval from the Animal Experiments Committee at the RIKEN Wako Institute (Approval number: H27-2-202).

### Pull-down assay

GST-tagged PIPKIα, PIPKIIα, and site-directed mutants of PIPKIα were expressed in E. coli (JM109 strain) using 100 μM isopropyl-β-D-thio-galactoside (IPTG) for 4 hr at 25°C, and purified with glutathione-Sepharose 4B (GE healthcare). The recombinant IRBIT protein expressed in Sf9 cells [3], the IP_3_ binding domain of IP_3_R1 (residues 224–604) fused to GST (GST-IP_3_BD) [2], and the N-terminal cytoplasmic domain of NBCe1-B (residues 1–468) fused to maltose-binding protein (MBP-NBCe1-B/N) [9] were described previously. For GST pull-down assay, cell lysates prepared as described above were incubated with 10 μg GST fusion proteins for 1 hr at 4°C. After the addition of 10 μl glutathione-Sepharose 4B, the samples were incubated for 1 hr at 4°C. The resins were washed three times with lysis buffer, and bound proteins were eluted with 20 mM glutathione. Pull-down assay using recombinant IRBIT protein (1 μg) were performed in pull-down buffer (10 mM Hepes [pH 7.4], 100 mM NaCl, 1 mM MgCl_2_, 1 mM EGTA, 1 mM 2-mercaptoethanol, and 0.01% Nonidet P-40) containing the indicated concentrations of PI4P, PI5P (CellSignals), IP_3_ (Dojindo), adenosine 5’-triphosphate (ATP), adenosine 5’-diphosphate (ADP), or thymidine 5’-triphosphate (TTP) (Sigma). The resins were washed three times with pull-down buffer. For MBP pull-down assay, cell lysates were incubated with 5 μg MBP fusion proteins for 1 hr at 4°C. After the incubation with 10 μl amylose resin (New England Biolabs) for 1 hr at 4°C, the resins were washed three times with lysis buffer, and bound proteins were eluted with 100 mM maltose. Eluted proteins were analyzed by immunoblotting with appropriate antibodies or Coomassie brilliant blue (CBB) staining.

### Immunoblotting

Samples were separated by SDS-PAGE and transferred onto polyvinylidene fluoride membrane (Millipore) by electroblotting. After blocking with 5% skim milk in PBS containing 0.1% Tween 20 (PBS-T) for 1 h at room temperature, membranes were immunoblotted with primary antibodies diluted in PBS-T containing 3% skim milk at the appropriate concentrations (anti-HA, 0.1 μg/ml; anti-myc conjugated with peroxidase, 0.5 μg/ml; other antibodies, 1 μg/ml) for 1 h at room temperature. After washing three times with PBS-T, the membrane was incubated with appropriate secondary antibodies conjugated with HRP diluted in PBS-T for 1 h at room temperature. When signals derive from goat antibodies used in the immunoprecipitation overlap those of immunoprecipitated proteins, the secondary antibody of the ImmunoCruz^TM^ IP/WB Optima D system (Santa Cruz Biotechnology) was used. After washing three times with PBS-T, immunoreactive bands were visualized with the ECL detection system (GE healthcare), and were captured using a luminescent image analyzer (LAS-4000 mini, GE healthcare). Band intensities were quantified using ImageJ software.

### Prediction of intrinsic disorder

Disordered/unstructured regions of IRBIT were analyzed by meta-prediction programs, MetaDisorder (http://genesilico.pl/metadisorder/, version MD2) [37], PONDR-FIT (http://www.disprot.org/pondr-fit.php) [38], and metaPrDOS (http://prdos.hgc.jp/cgi-bin/meta/top.cgi) [39].

### Measurement of PIPK activity

In vitro lipid kinase assays were performed as described previously [32, 36] with modifications. Lysates of transfected COS-7 cells or MEF cells were incubated with 2 μg appropriate antibodies for 2 hr at 4°C. The immune complex was isolated by the addition of 10 μl protein G-Sepharose beads for 1hr at 4°C. The complex was spun down and washed three times with lysis buffer and three times with reaction buffer (50 mM Tris-HCl [pH 7.5], 100 mM NaCl, 1mM MgCl_2_, and 1mM EGTA). For PIPKIα activity, the reaction was started by adding 50 μM PI4P, 50 μM ATP, and 10 μCi of [γ-^32^P]ATP (PerkinElmer Life Sciences) in 50 μl. For PIPKIIα activity, 50 μM PI5P was used instead of PI4P. After incubation for 10–20 min at 37°C, the lipids were extracted with chloroform/methanol and spotted onto TLC plates (Merck). The plates were developed in chloroform/methanol/ammonia/water, and the products were observed by autoradiography. PI(4,5)P_2_ production was quantified by a liquid scintillation counter (Packard/PerkinElmer).

### Immunocytochemistry

Transfected HeLa cells grown on glass coverslips were washed once with PBS, fixed in 4% formaldehyde in PBS for 15 min, permeabilized in 0.1% Triton X-100 in PBS for 5 min, and blocked in PBS containing 2% normal goat serum (Vector Laboratories) for 60 min at room temperature. Cells were then stained with rabbit anti-HA (1 μg/ml) and mouse anti-myc (1 μg/ml) for 60 min at room temperature. Following four 5-min PBS washes, Alexa Fluor 594-conjugated goat anti-rabbit IgG (1 μg/ml) and Alexa Fluor 488 or 647-conjugated goat anti-mouse IgG (1 μg/ml) were applied for 45 min at room temperature. Following four 5-min PBS washes, the coverslips were mounted with Vectashield (Vector Laboratories) and observed under a confocal fluorescence microscopy (FV1000, Olympus) with a ×60 objective. Fluorescence images were analyzed by FV10-ASW software (Olympus). The average fluorescence intensity of the two cell edges were defined as the plasma membrane and regions between the cell edges were defined as cytosol.

## Results and Discussion

### IRBIT interacts with PIPKIα and PIPKIIα in cerebellum

**Fig 1:**
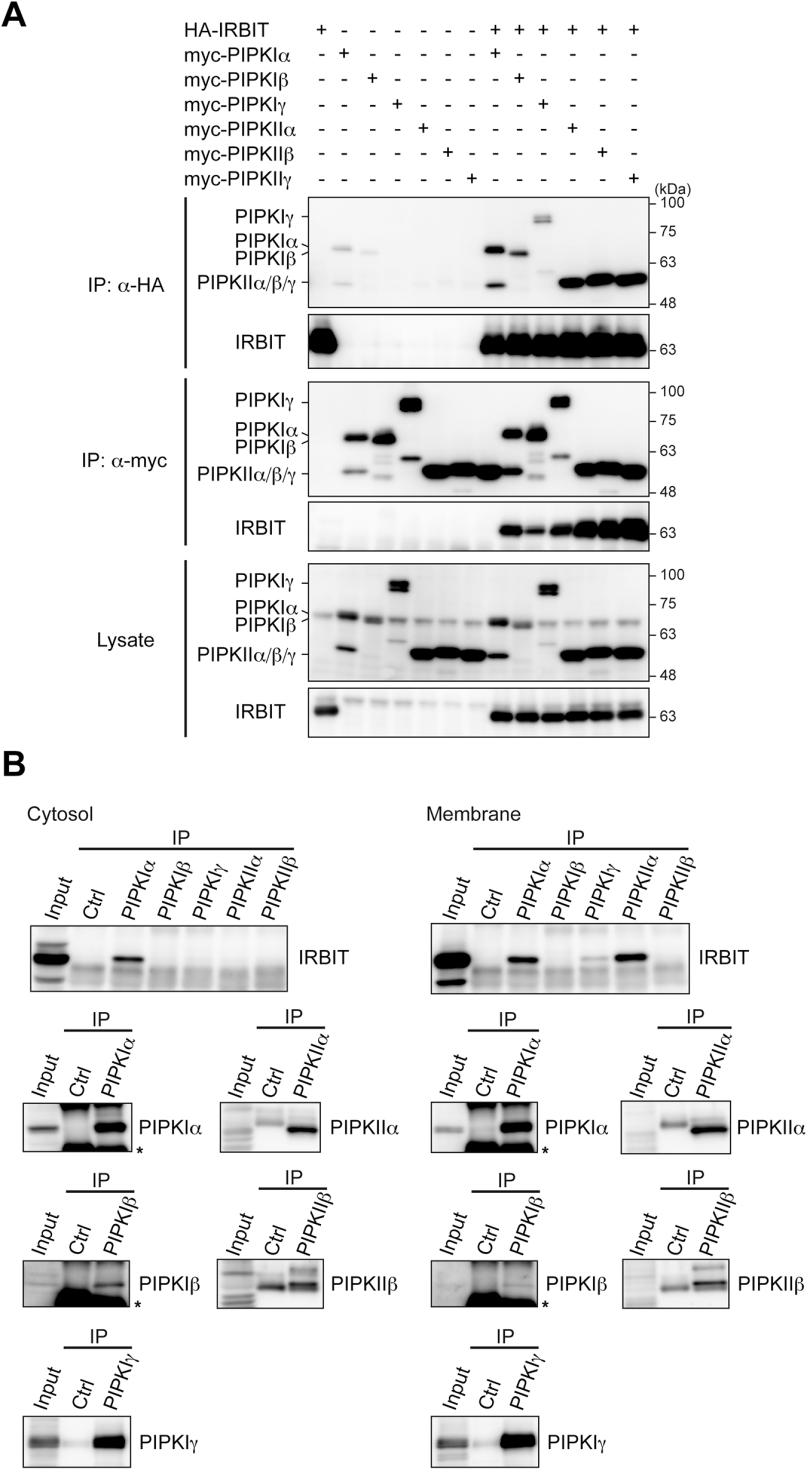
IRBIT interacts with PIPKIα and PIPKIIα in mouse cerebellum. (A) Interaction between IRBIT and PIPK family members in heterologous cells. HA-IRBIT and myc-PIPK isoforms transfected into COS-7 cells were immunoprecipitated with anti-HA or anti-myc antibody. Immunoprecipitates were analyzed by Western blotting with anti-HA or anti-myc antibody. (B) Interaction between IRBIT and PIPK family members in mouse cerebellum. Cytosolic and membrane fractions were processed for immunoprecipitation with antibodies specific to each PIPK isoform or control (Ctrl) antibody, and immunoprecipitates were analyzed by Western blotting with antibodies indicated. Anti-PIPKIIγ antibody that successfully precipitates PIPKIIγ was not available. Asterisks indicate immunoglobulin heavy chains.

We previously reported proteomic analysis of IRBIT-interacting proteins, where IRBIT was overexpressed in 293EBNA cells and proteins immunoprecipitated with IRBIT were analyzed by mass spectrometry [20]. Among the proteins identified was a protein belonging to the PIPK family, PIPKIIγ [32]. Here we investigated the interaction of IRBIT with the PIPK isoforms PIPKIα, PIPKIβ, PIPKIγ, PIPKIIα, PIPKIIβ, and PIPKIIγ (human nomenclature is used in this study). IRBIT and each isoform of the PIPK family were transfected into COS-7 cells, and co-immunoprecipitation assays were performed. IRBIT interacted with all PIPK isoforms in the heterologous expression system (Fig 1A, S1 Fig).

Further, we tested the interaction of IRBIT with PIPK family members in vivo. Mouse cerebella, where IRBIT is highly expressed [2, 40], were fractionated into cytosolic and membrane fractions and processed for immunoprecipitation using antibodies specific to each PIPK isoform. IRBIT was clearly detected in the anti-PIPKIα immunoprecipitates from the cytosol fraction and in the anti-PIPKIα and anti-PIPKIIα immunoprecipitates from the membrane fraction (Fig 1B, S1 Fig). Faint evidence of IRBIT was observed in the anti-PIPKIγ immunoprecipitates from the membrane fraction (Fig 1B, S1 Fig). These results indicate that isoform specificity exists in vivo, and IRBIT mainly binds to PIPKIα and PIPKIIα in mouse cerebellum. The restriction of IRBIT/PIPKIIα interaction to the membrane fraction might be accounted for by the observation that IRBIT is more highly phosphorylated in membrane than in cytosolic fractions [40]. Although these results do not exclude the possibility that IRBIT interacts with other PIPK isoforms in different tissues, we hereafter focused on its interaction with PIPKIα and PIPKIIα.

### Conserved catalytic amino acids of PIPKIα and PIPKIIα are required for interaction with IRBIT

**Fig 2:**
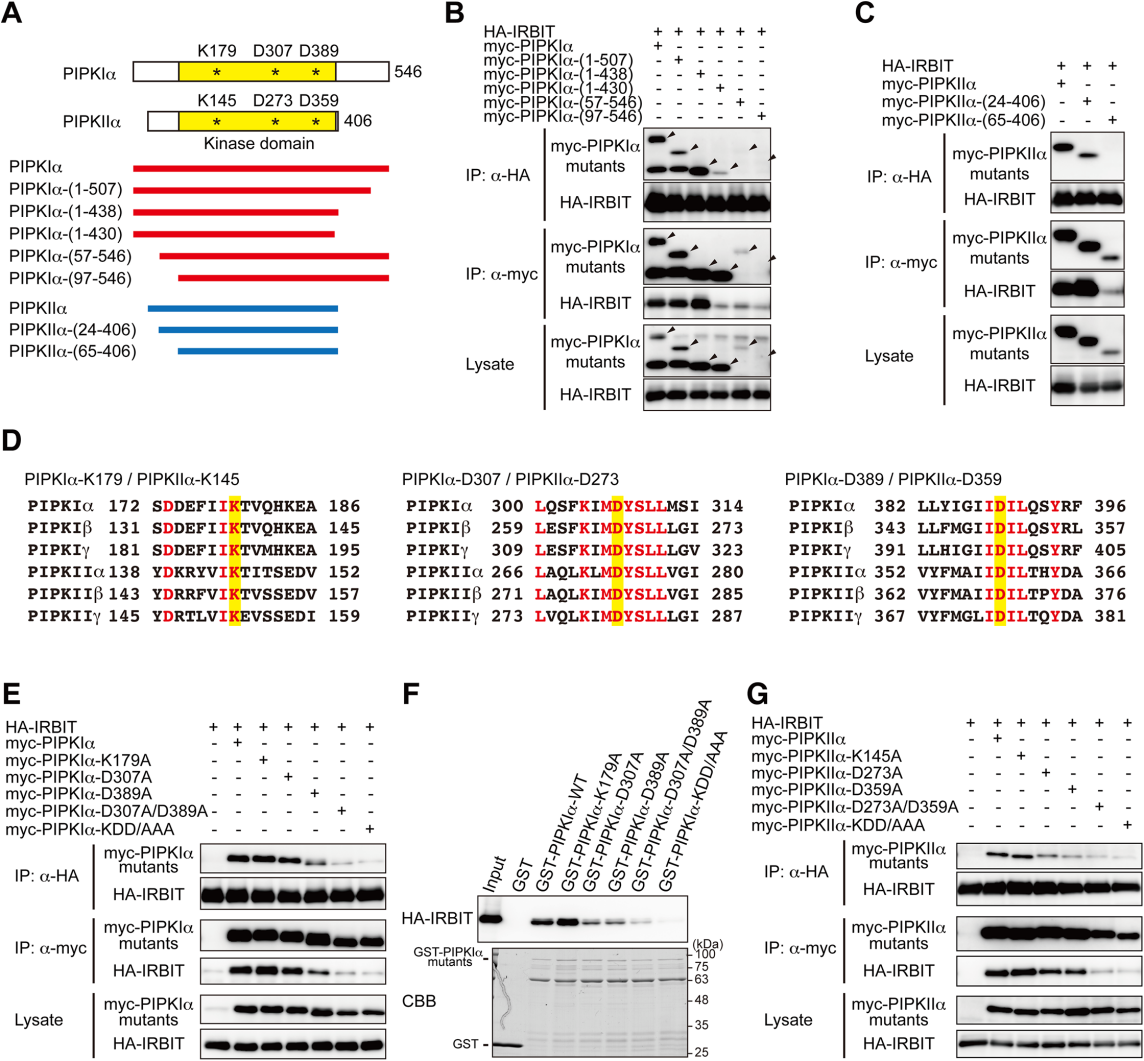
Conserved catalytic amino acids of PIPKIα and PIPKIIα are required for interaction with IRBIT. (A) Diagram of deletion mutants of PIPKIα and PIPKIIα. Kinase domains are indicated by yellow boxes. Catalytic amino acids are indicated by asterisks. (B, C, E, G) Immunoprecipitation between IRBIT and PIPKIα/PIPKIIα mutants. HA-IRBIT and deletion mutants (B, C) or site-directed mutants (E, G) of myc-PIPKIα (B, E) or myc-PIPKIIα (C, G) were transfected into COS-7 cells and immunoprecipitated with anti-HA or anti-myc antibody. Immunoprecipitates were analyzed by Western blotting with anti-HA or anti-myc antibody. The sizes of PIPKIα deletion mutants are indicated by arrowheads in (B). KDD/AAA indicate the triple mutants. (D) Amino acid sequences of catalytic residues of PIPK family members. Catalytic amino acids are highlighted in yellow. Amino acids perfectly conserved among PIPK family members are indicated by red letters. (F) Pull-down of HA-IRBIT overexpressed in COS-7 cells using site-directed mutants of GST-PIPKIα. Bound proteins were analyzed by Western blotting with anti-HA (top) and CBB staining (bottom).

PIPK family members have a conserved kinase homology domain and divergent N- and C-terminal regions [25, 27] (Fig 2A). To identify the regions responsible for binding to IRBIT, we analyzed the interaction of IRBIT with deletion mutants of PIPKIα and PIPKIIα. The C-terminal deletion mutants PIPKIα-(1–507) and PIPKIα-(1–438) bound to IRBIT, whereas PIPKIα-(1–430) showed significantly decreased affinity to IRBIT (Fig 2A and 2B). The expression levels of the N-terminal truncation mutants, PIPKIα-(57–546) and PIPKIα-(97–546), were much lower than that of wild-type PIPKIα (Fig 2A and 2B). These results suggest that amino acids 431–438 of PIPKIα are critical for the interaction with IRBIT, and the N-terminal divergent regions are essential for the stability of PIPKIα. Deletion of the N-terminal region of PIPKIIα resulted in low expression and reduced interaction with IRBIT (Fig 2A and 2C).

Although the homology of primary sequences of the kinase domains of type I and type II PIPKs is not very high (30%–40% identity) [27–32], their 3D structures are assumed to be well conserved [41, 42]. Considering that all PIPK isoforms bound to IRBIT in a heterologous expression system (Fig 1A, S1 Fig), the conserved kinase homology domain is expected to be involved in the interaction with IRBIT. Our previous study showed that IRBIT binds to the IP_3_-binding core of IP_3_R via amino acids essential for IP_3_ recognition [2, 3]. Similarity in chemical structures between IP_3_ and the PIPK substrates, PI4P and PI5P, both having an inositol ring and two or three phosphoryl groups, prompted us to investigate whether IRBIT interacts with the catalytic core of PIPKIα and PIPKIIα. Structural homologies between PIPK family members and protein kinases and mutagenesis analysis showed that Lys179, Asp307, and Asp389 of PIPKIα, and the corresponding Lys145, Asp273, and Asp359 of PIPKIIα are located in the catalytic core and are essential for enzymatic activity [29, 41–43] (Fig 2A). These residues are completely conserved among PIPK family members (Fig 2D). We introduced mutations in Lys179, Asp307, and Asp389 of PIPKIα and tested their interaction with IRBIT. Co-immunoprecipitation assays revealed that the D389A mutant displayed slightly decreased affinity for IRBIT, and that the double mutation D307A/D389A further decreased the interaction with IRBIT (Fig 2E). To exclude the indirect effect of actin re-organization induced by PIPKIα overexpression [44] on this interaction, we performed pull-down assays using recombinant GST fusion proteins of PIPKIα mutants. We found that the D307A and D389A mutants exhibited reduced binding to IRBIT, and the double mutant D307A/D389A displayed further diminished interactions (Fig 2F). Similar results were obtained for PIPKIIα. Mutation of Asp273 or Asp359 of PIPKIIα, corresponding to Asp307 and Asp389 of PIPKIα, respectively, slightly decreased PIPKIIα binding to IRBIT, and the double mutation D273A/D359A markedly weakened this interaction (Fig 2G). Thus, aspartate residues, Asp307/Asp389 and Asp273/Asp359, which are critical for the kinase activity of PIPKIα and PIPKIIα, respectively, are essential for interaction with IRBIT. These results suggest that IRBIT interacts with the catalytic core of PIPKIα and PIPKIIα, as with their phosphatidylinositol phosphate substrates.

**Fig 3:**
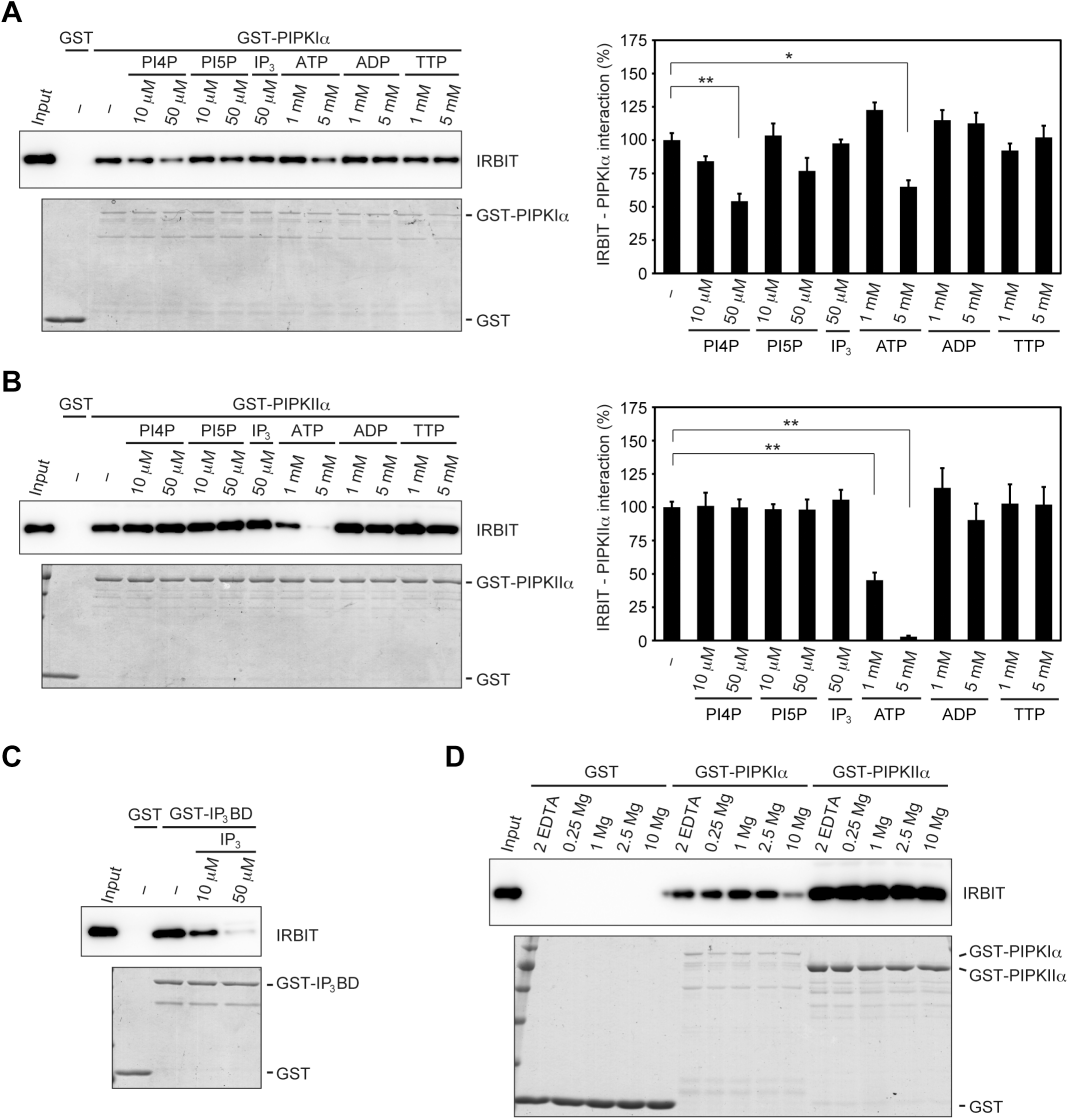
PI4P, ATP, or Mg^2+^ interferes with the binding of IRBIT to PIPKIα and PIPKIIα. (A, B, C) Recombinant IRBIT protein was processed for pull-down assay using GST-PIPKIα (A), GST-PIPKIIα (B), or GST-IP_3_BD (C) in the presence of the indicated concentrations of PI4P, PI5P, IP_3_, ATP, ADP, or TTP. Bound proteins were analyzed by Western blotting with anti-IRBIT (top) and CBB staining (bottom). The relative binding of IRBIT to PIPKIα and PIPKIIα are presented as mean +/- SEM (*n* = 3–4). * *p* < 0.05, ** *p* < 0.01 (one-way ANOVA with post-hoc Tukey-Kramer test). (D) IRBIT was pulled down with GST, GST-PIPKIα, or GST-PIPKIIα in the presence of 2 mM EDTA or 0.25, 1, 2.5, or 10 mM Mg^2+^.

To clarify further the possibility that IRBIT binds to the catalytic core of PIPKs, we tested whether the PIPK substrates, ATP, and Mg^2+^, which interact with the catalytic core of PIPKs, interfere with the interaction between IRBIT and PIPKIα/IIα. Pull-down assay using recombinant IRBIT showed that the binding of IRBIT to PIPKIα was suppressed by PI4P and 5 mM ATP, but not by PI5P, IP_3_, ADP, and TTP (Fig 3A). On the other hand, the interaction between IRBIT and PIPKIIα was efficiently inhibited by 1–5 mM ATP, but not by PI4P, PI5P, IP_3_, ADP, and TTP, in a dose-dependent manner (Fig 3B). As a control, IP_3_ disrupted the interaction between IRBIT and the IP_3_ binding domain (IP_3_BD) of IP_3_R (Fig 3C), as reported previously [2, 3]. A high concentration of Mg^2+^ (10 mM) reduced the binding of IRBIT to PIPKIα, but not to PIPKIIα, although physiological concentrations of Mg^2+^ (0.25–1 mM) [45] did not affect the interactions (Fig 3D). These results indicate that substances that bind to the catalytic core of PIPKs interfere with the IRBIT binding, supporting the hypothesis that IRBIT interacts with the catalytic core of PIPKIα and PIPKIIα. The mechanism of the interactions is different between PIPKIα and PIPKIIα, that is, the PIPKIα-IRBIT interaction is sensitive to PI4P, ATP, and Mg^2+^, whereas the PIPKIIα-IRBIT interaction is resistant to PI4P/PI5P and Mg^2+^, and is more sensitive to ATP than the PIPKIα-IRBIT interaction. Different affinities of the interactions and/or involvements of non-conserved amino acids in the catalytic core of PIPKIα and PIPKIIα might explain this difference. Intracellular ATP levels are approximately 1 mM in mammalian cells [46–48] and can be increased to several millimoles by extracellular stimuli [46, 49]. Thus, the interaction between IRBIT and PIPKs might be regulated by changes of cytosolic ATP concentrations.

Several lines of evidence suggest that IRBIT behaves as if its moiety mimics the structure or function of inositol phosphates or phosphoinositides. First, IRBIT binds to the IP_3_-binding core of IP_3_R by recognizing amino acids that interact with IP_3_ [3, 50]. Second, IRBIT and PI(4,5)P_2_ bind to the same domain of NBCe1-B and activate NBCe1-B, in part by a common mechanism [13]. Third, IRBIT binds to PIPKIα and PIPKIIα via amino acids that catalyze phosphatidylinositol phosphates (Fig 2D–2G), and PI4P inhibits the IRBIT-PIPKIα interaction (Fig 3A). These findings suggest that phosphorylated serine/threonine residues of IRBIT (discussed below) simulate phosphate groups of inositol phosphates and phosphoinositides.

### Phosphorylation sites of IRBIT are essential for its interaction with PIPKIα and PIPKIIα

**Fig 4:**
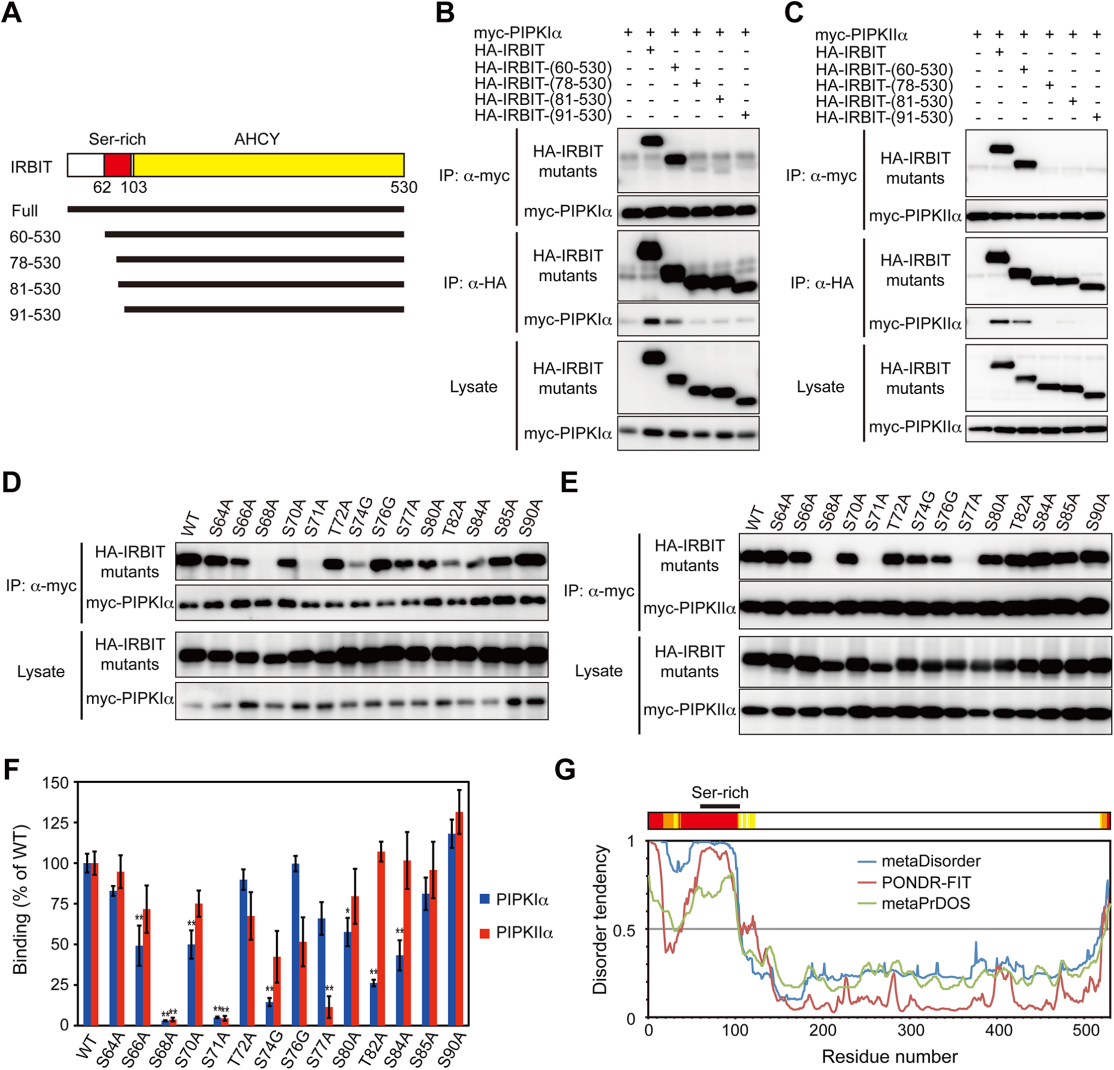
Phosphorylation sites in the serine-rich region of IRBIT are essential for its interaction with PIPKIα and PIPKIIα. (A) The structure of IRBIT. The serine-rich region (red) and the AHCY domain (yellow) are indicated. (B-E) Interaction between deletion mutants (B, C) or site-directed mutants (D, E) of IRBIT and PIPKIα (B, D) or PIPKIIα (C, E). HA-IRBIT mutants were transfected with myc-PIPKIα or myc-PIPKIIα into COS-7 cells and were processed for immunoprecipitation with anti-HA or anti-myc antibody. Immunoprecipitates were analyzed by Western blotting with anti-HA or anti-myc antibody. (F) Quantification of (D) and (E). The relative binding of site-directed mutants of IRBIT to PIPKIα and PIPKIIα are presented as mean +/- SEM (*n* = 4). * *p* < 0.05, ** *p* < 0.01, compared with wild-type (WT) IRBIT (one-way ANOVA with post-hoc Tukey’s HSD test). (G) Prediction of intrinsically disordered regions of IRBIT. Amino acid sequence of IRBIT was analyzed by three different meta-protein disorder prediction programs, metaDisorder [37], PONDR-FIT [38], and metaPrDOS [39]. Disordered regions predicted by three (red), two (orange), or one (yellow) programs are indicated.

IRBIT comprises an AHCY domain and an N-terminal extension containing a serine-rich region [2, 6] (Fig 4A). The serine-rich region (residues 62–103) is subject to multiple phosphorylations [3, 7, 8] that regulate IRBIT interactions with target proteins [3, 7, 9, 16, 17, 20, 21]. To clarify the involvement of the serine-rich region in the binding to PIPKs, we analyzed the interaction of IRBIT N-terminal deletion mutants with PIPKIα and PIPKIIα. IRBIT-(60–530) bound to both PIPKIα and PIPKIIα; however, IRBIT-(78–530) almost completely lost the ability to bind PIPKIα and PIPKIIα (Fig 4B and 4C), suggesting that critical binding determinants are located in the serine-rich region of IRBIT.

Several serine/threonine residues in the serine-rich region of IRBIT are phosphorylated. Ser68 is phosphorylated by protein kinase D [3, 7, 17]. Once Ser68 is phosphorylated, Ser71, Ser74, and probably Ser77 are subsequently phosphorylated by casein kinase I [3, 7]. Thr82, Ser84, and Ser85 are phosphorylated in mouse brain [8]. Thus, we analyzed the interaction of site-directed mutants of serine/threonine residues in the IRBIT serine-rich region with PIPKIα and PIPKIIα. Mutation of Ser68 or Ser71 had the most prominent effect, showing almost complete loss of interaction with both PIPKIα and PIPKIIα (Fig 4D–4F). Mutation of Ser74, Thr82, or Ser84 markedly reduced the binding to PIPKIα, but had little effect on the binding to PIPKIIα. Mutation of Ser66, Ser70, or Ser80 also significantly decreased the interaction with PIPKIα, but not with PIPKIIα. In contrast, mutation of Ser77 profoundly decreased binding to PIPKIIα, but had less effect on the interaction with PIPKIα (Fig 4D–4F). Thus, Ser68 and Ser71 are commonly required for the interaction with PIPKIα and PIPKIIα, and Ser66/Ser70/Ser74/Ser80/Thr82/Ser84 and Ser77 are specifically important for the binding to PIPKIα and PIPKIIα, respectively. These results suggest that the phosphorylation status of the serine-rich region determines the binding specificity of IRBIT to PIPK isoforms. Furthermore, differences in the phosphorylation status of IRBIT between the cytosol and membrane fractions [2, 40] may explain the result that IRBIT bound to PIPKIIα in the membrane fraction but not in the cytosolic fraction (Fig 1B, S1 Fig).

### The N-terminal region of IRBIT is intrinsically highly disordered

The serine-rich region of IRBIT is critically involved in its interaction with most of its binding partners [2, 3, 9, 10, 16, 17, 20–22]. The molecular mechanisms by which the serine-rich region interacts with diverse proteins that have no sequence or structural homology remain largely unknown. Circular dichroism spectroscopy and limited proteolysis analyses suggest that the N-terminal domain of IRBIT has a loose structure [51]. In addition, the crystal structure of IRBIT available in the Protein Data Bank (PDB number 3MTG) lacks the N-terminal 100 amino acids [52]. These observations suggest that the regions surrounding the serine-rich region have flexible structures. Thus, we investigated the structural order/disorder of IRBIT using meta-protein disorder prediction programs [37–39]. The in silico predictions revealed that the N-terminal approximately 100 amino acids of IRBIT containing the serine-rich region is highly disordered (Fig 4G), indicating that the N terminus of IRBIT belongs to the intrinsically disordered protein (IDP) regions [53].

The IDP regions do not form stable structures, and their instability is encoded by amino acid sequences [53]. The conformational flexibility of the IDP regions is beneficial for interactions with diverse proteins [54]. The structure and function of the IDP regions is regulated by phosphorylation [55, 56]. The structural flexibility of the serine-rich region of IRBIT may underlie the mechanism of its interactions with multiple divergent proteins and its regulation by phosphorylation, and may allow for its intrusions into the catalytic core of PIPKs (Fig 2D–2G) and the IP_3_ binding core of IP_3_R [3]. The IRBIT gene is assumed to emerge by gene duplication of an ancestral AHCY gene in the course of evolution [6]. The key roles of the serine-rich region in IRBIT function suggest that acquisition of the N-terminal IDP region confers IRBIT with crucial abilities to regulate diverse cellular processes required in higher organisms.

### IRBIT does not regulate the kinase activity of PIPKIα and PIPKIIα

**Fig 5:**
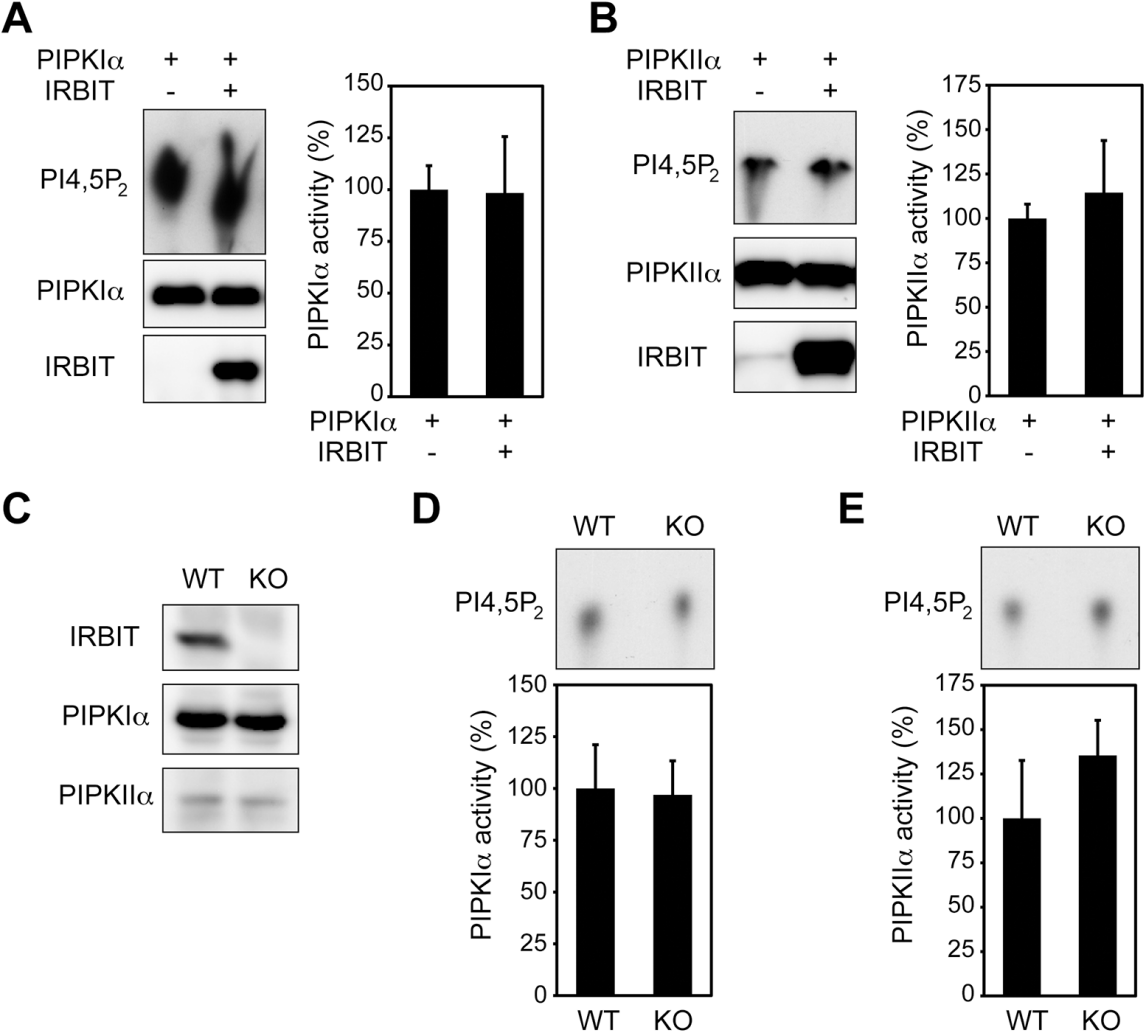
IRBIT does not regulate the kinase activity of PIPKIα and PIPKIIα. (A) Effect of IRBIT on the kinase activity of PIPKIα. myc-PIPKIα was transfected with or without pcDNA3-IRBIT into COS-7 cells and immunoprecipitated with anti-myc antibody. Ninety percent of immunoprecipitates were incubated with PI4P and [^32^P]ATP, and the [^32^P]PI(4,5)P_2_ production was measured. Ten percent of immunoprecipitates were analyzed by Western blotting with anti-myc or anti-IRBIT antibody. (B) Effect of IRBIT on the kinase activity of PIPKIIα. myc-PIPKIIα activity was measured as described in (A). PI5P was used as a substrate instead of PI4P. (C) MEF cells derived from wild-type (WT) or IRBIT knockout (KO) mice were analyzed by Western blotting with anti-IRBIT, anti-PIPKIα, or anti-PIPKIIα antibody. (D) PIPKIα activity in IRBIT-KO cells. MEF cell lysates were processed for immunoprecipitation with anti-PIPKIα antibody. Immunoprecipitates were incubated with PI4P and [^32^P]ATP, and the [^32^P]PI(4,5)P_2_ production was measured. (E) PIPKIIα activity in IRBIT-KO cells. Immunoprecipitates with anti-PIPKIIα were incubated with PI5P and [^32^P]ATP, and the [^32^P]PI(4,5)P_2_ production was measured. Bar graphs in A, B, D, and E represent quantification of PIP_2_ production. Data are presented as means +/- SD (*n* = 3). No statistically significant differences were detected (Student’s t-test).

We tested whether IRBIT regulates the enzymatic activity of PIPKIα and PIPKIIα. In vitro production of PI(4,5)P_2_ was measured using immunoprecipitates from COS-7 cells transfected with PIPKIα with or without IRBIT. IRBIT had no effect on the kinase activity of PIPKIα (Fig 5A). Similar results were obtained for PIPKIIα (Fig 5B). Further, we analyzed the activity of PIPKIα and PIPKIIα in MEF cells derived from IRBIT knockout mice [22]. Expression of PIPKIα and PIPKIIα did not differ between wild-type and IRBIT knockout MEF cells (Fig 5C). In vitro lipid kinase assays of immunoprecipitates with anti-PIPKIα or anti-PIPKIIα antibodies showed that IRBIT knockout did not influence PI(4,5)P_2_ production (Fig 5D and 5E). These results suggest that IRBIT does not regulate the lipid kinase activity of PIPKIα and PIPKIIα.

The interaction of IRBIT with the catalytic core of PIPKIα and PIPKIIα (Fig 2D–2G, Fig 3) may account for the inability of IRBIT to regulate the enzymatic activity of PIPKs. That is, the PI4P and/or ATP used in the in vitro lipid kinase assays might competitively disrupt the interaction between IRBIT and PIPKs. Therefore, we cannot exclude the possibility that IRBIT might regulate PIPK activity when the substrate concentration is extremely low.

### IRBIT and PIPKs interact with NBCe1-B

**Fig 6:**
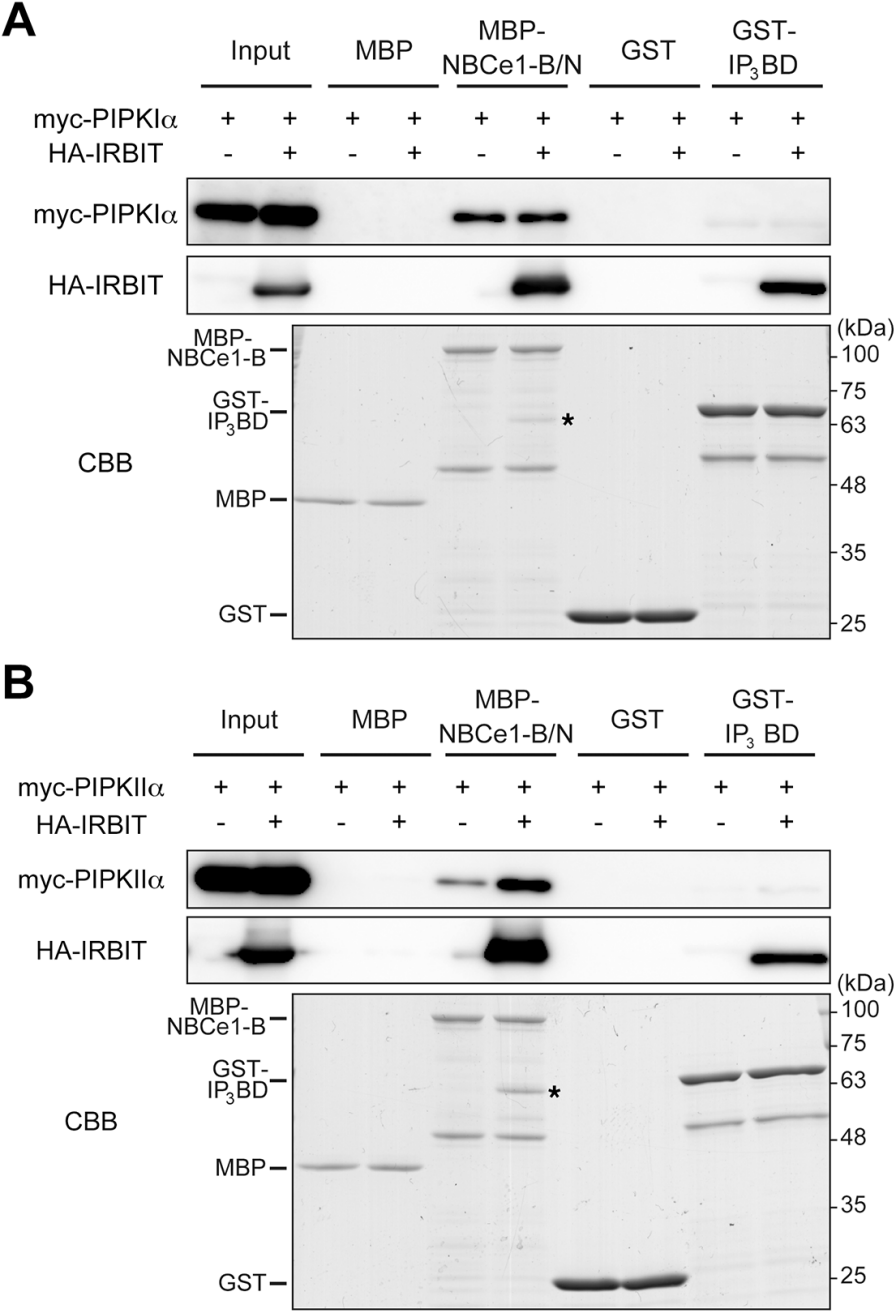
PIPKIα and PIPKIIα interact with NBCe1-B. Lysates of COS-7 cells overexpressing myc-PIPKIα (A) or myc-PIPKIIα (B) with or without HA-IRBIT were incubated with MBP, MBP-NBCe1-B/N, GST, or GST-IP_3_BD. Bound proteins were subjected to Western blotting with anti-myc (top) and anti-HA (middle), and CBB staining (bottom). Asterisks indicate HA-IRBIT pulled down with MBP-NBCe1-B/N.

We tested whether IRBIT and PIPKs form complexes with PI(4,5)P_2_ effector proteins. IRBIT interacts with and activates the Na^+^/HCO_3_^−^ cotransporter NBCe1-B [9–14], an ion transporter that mediates Na^+^-dependent HCO_3_^−^ transport across the plasma membrane [57]. NBCe1-B activity is regulated by PI(4,5)P_2_ [13, 35]. Thus, we examined the binding of PIPKIα to NBCe1-B by pull-down assay using the N-terminal cytoplasmic domain of NBCe1-B (NBCe1-B/N) [9]. Further, we tested the interaction of PIPKIα with IP_3_R using the IP_3_ binding domain (IP_3_BD) of IP_3_R [2], as IP_3_, the agonist of IP_3_R, is generated by the hydrolysis of PI(4,5)P_2_. As shown in Fig 6A, PIPKIα interacted with NBCe1-B/N but not with IP_3_BD, although IRBIT bound to both NBCe1-B/N and IP_3_BD, as reported previously [2, 9]. These results suggest that IRBIT, PIPKIα, and NBCe1-B form a ternary complex. Further, we examined the interaction of PIPKIIα with NBCe1-B or IP_3_R. IRBIT enhanced the interaction of PIPKIIα with NBCe1-B/N but not with IP_3_BD (Fig 6B), suggesting that IRBIT recruits PIPKIIα to NBCe1-B.

**Fig 7:**
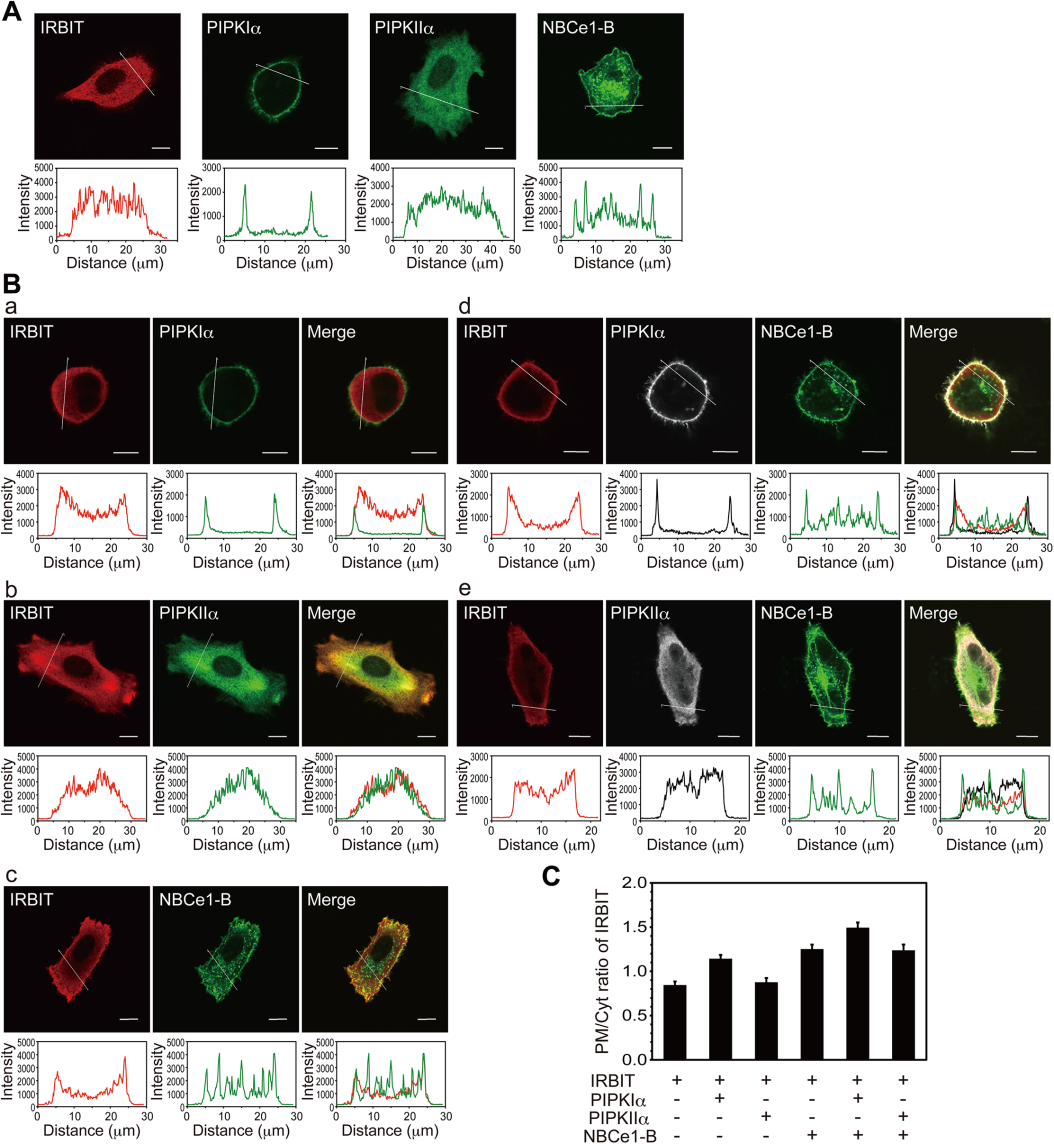
IRBIT colocalizes with PIPKIα and NBCe1-B at the plasma membrane in HeLa cells. (A) HA-IRBIT, myc-PIPKIα, or myc-PIPKIIα were transfected into HeLa cells and immunostained with anti-HA or anti-myc antibody. GFP-NBCe1-B was transfected into HeLa cells and GFP fluorescence was observed. (B) HA-IRBIT was transfected into HeLa cells with myc-PIPKIα (a), myc-PIPKIIα (b), GFP-NBCe1-B (c), myc-PIPKIα and GFP-NBCe1-B (d), or myc-PIPKIIα and GFP-NBCe1-B (e). Cross-sectional plots of fluorescence intensities are shown below the images. Scale bars, 10 μm. (C) Plasma membrane/cytosol ratios of IRBIT fluorescence intensity. Mean +/- SEM are shown (28–33 cells from three independent experiments).

Next, we investigated intracellular localization of IRBIT, PIPKIα, PIPKIIα, and NBCe1-B in HeLa cells. When overexpressed alone, IRBIT and PIPKIIα were localized in the cytosol, whereas PIPKIα was localized at the plasma membrane (Fig 7A). Cells overexpressing PIPKIα showed a rounded cell shape due to actin re-organization induced by PI(4,5)P_2_ production [29, 44] (Fig 7A). NBCe1-B was localized at the plasma membrane and intracellular vesicles (Fig 7A). When IRBIT was co-expressed with PIPKIα, or NBCe1-B, but not with PIPKIIα, IRBIT was partially localized near the plasma membrane (Fig 7B and 7C). IRBIT co-localized with NBCe1-B at the plasma membrane, but not on intracellular vesicles (Fig 7Bc). The effects of PIPKIα and NBCe1-B on the plasma membrane targeting of IRBIT might be additive (Fig 7B and 7C). IRBIT, PIPKIα, and NBCe1-B colocalized at the plasma membrane (Fig 7Bd). These results suggest that IRBIT, PIPKIα, and NBCe1-B form signaling complexes at the plasma membrane.

The physiological role of the complexes containing IRBIT, PIPKIα and NBCe1-B is unclear. IRBIT directly activates NBCe1-B [9–14], whereas PIPKIα may indirectly potentiates the activity of NBCe1-B through the production of PI(4,5)P_2_ in close proximity to NBCe1-B. IRBIT is most highly expressed in the brain [2, 40], and IRBIT knockout mice show behavioral abnormities such as hyperactivity and increased social interactions [22]. PIPKIα is abundantly expressed in the brain and is responsible for the generation of specific PI4,5,P_2_ pools [58]. In the brain, NBCe1-B plays a crucial role in the regulation of intracellular pH in neurons and astrocytes [59–62]. In fact, human patients with mutations in the NBCe1 gene (*SLC4A4*) show neurological abnormalities such as mental retardation and migraines [60, 63–66]. Thus, the signaling complex containing IRBIT, PIPKIα, and NBCe1-B may play an important role in the central nervous system. Compared to type I PIPKs, type II PIPKs play a minor role in PI(4,5)P_2_ production, as the basal level of PI5P is only 0.5%–2% that of PI4P [67]. Therefore, the major function of type II PIPKs may be to control PI5P levels [68]. PI5P is the least characterized of the phosphoinositides; however, one of its functions is speculated to be the regulation of intracellular vesicle trafficking [68–70]. Thus, the IRBIT-PIPKIIα complex may regulate NBCe1-B trafficking. In the cerebellum, IRBIT bound to PIPKIIα in the membrane fraction, but not in the cytosol (Fig 1B, S1 Fig); however, IRBIT colocalized with cytosolic PIPKIIα in HeLa cells (Fig 7Bb). This discrepancy may be explained by the abundant expression of NBCe1-B and its splicing variant, NBCe1-C, in brain [59, 60], and/or difference in phosphorylation status of IRBIT between brain and HeLa cells. Further studies are required to reveal physiological roles of the interaction between IRBIT, PIPKIα/IIα, and NBCe1-B.

## Conclusions

In this study, we demonstrated that IRBIT interacts with PIPKs, lipid kinases that generate PI(4,5)P_2_. Specifically, IRBIT bound to PIPKIα and PIPKIIα isoforms in the cerebellum. Mutagenesis analysis and inhibition of the interaction by PI4P, Mg^2+^ and/or ATP suggest that IRBIT interacts with the catalytic core of PIPKIα and PIPKIIα through conserved catalytic aspartate residues. Phosphorylation sites in the serine-rich region of IRBIT are essential for the selectivity of its interaction with PIPKIα and PIPKIIα, suggesting the possibility that phosphorylated serine/threonine residues of IRBIT might simulate phosphate groups of phosphatidylinositol phosphate substrates. The structural flexibility of the N-terminal region of IRBIT may underlie the mechanism of its interactions with multiple divergent proteins. Furthermore, in vitro binding experiments and immunocytochemistry suggest that IRBIT and PIPKIα interact with the Na^+^/HCO_3_^−^ cotransporter NBCe1-B. These results suggest that IRBIT forms signaling complexes with PIPKIα and NBCe1-B, whose activity is regulated by PI(4,5)P_2_.

## Supporting Information

S1 Fig IRBIT interacts with PIPKs in the presence of 1 mM Mg^2+^. (A) Immunoprecipitation from heterologous cells using the lysis buffer containing 1 mM MgCl_2_ and 1 mM EGTA instead of 2 mM EDTA. HA-IRBIT and myc-PIPK isoforms transfected into COS-7 cells were immunoprecipitated with anti-HA or anti-myc antibody. Immunoprecipitates were analyzed by Western blotting with anti-HA or anti-myc antibody. (B) Immunoprecipitation from mouse cerebellum using buffers containing 1 mM MgCl_2_ and 1 mM EGTA instead of 2 mM EDTA. Cytosolic and membrane fractions were processed for immunoprecipitation with antibodies specific to each PIPK isoform or control (Ctrl) antibody, and immunoprecipitates were analyzed by Western blotting with antibodies indicated. Asterisks indicate immunoglobulin heavy chains.
(TIF)
