# Supplementary figures and images for: IRBIT Interacts with the Catalytic Core of Phosphatidylinositol Phosphate Kinase Type Iα and IIα through Conserved Catalytic Aspartate Residues
Source: PLoS One. 2015 Oct 28;10(10):e0141569. doi: 10.1371/journal.pone.0141569 (PMC4624786; doi:10.1371/journal.pone.0141569)

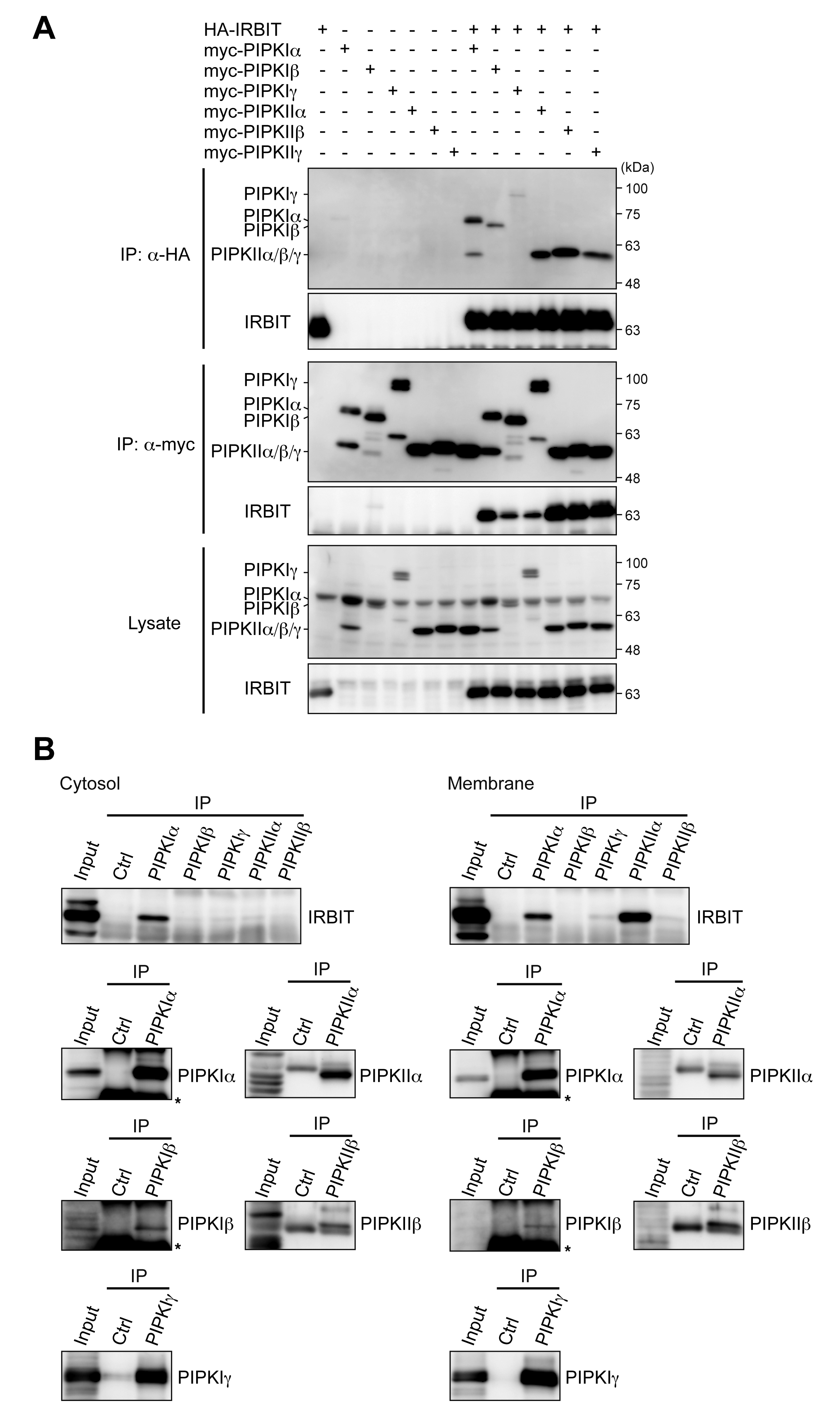

Supplement: S1 Fig — (A) Immunoprecipitation from heterologous cells using the lysis buffer containing 1 mM MgCl2 and 1 mM EGTA instead of 2 mM EDTA. HA-IRBIT and myc-PIPK isoforms transfected into COS-7 cells were immunoprecipitated with anti-HA or anti-myc antibody. Immunoprecipitates were analyzed by Western blotting with anti-HA or anti-myc antibody. (B) Immunoprecipitation from mouse cerebellum using buffers containing 1 mM MgCl2 and 1 mM EGTA instead of 2 mM EDTA. Cytosolic and membrane fractions were processed for immunoprecipitation with antibodies specific to each PIPK isoform or control (Ctrl) antibody, and immunoprecipitates were analyzed by Western blotting with antibodies indicated. Asterisks indicate immunoglobulin heavy chains. (TIF) [file pone.0141569.s001.tif]
